# Discrepancy in PD-L1 expression between primary and metastatic tumors in two patients with recurrent cervical cancer

**DOI:** 10.1016/j.gore.2024.101484

**Published:** 2024-08-12

**Authors:** Brittany File, Anjali Hari

**Affiliations:** a Department of Obstetrics and Gynecology, University of California, Irvine Medical Center, Orange, CA, USA; b Department of Obstetrics and Gynecology, Division of Gynecologic Oncology, University of California, Irvine Medical Center, Orange, CA, USA

**Keywords:** PD-L1 expression, CPS Score, Recurrent cervical cancer, Pembrolizumab eligibility, Discrepancies in expression

## Abstract

**Introduction:**

Pembrolizumab is an immunotherapy approved for use in patients with a combined positive score (CPS) greater than one with recurrent cervical cancer. In clinical practice, the CPS score is not typically analyzed in both primary and metastatic specimens.

**Case descriptions:**

Case 1

A 42-year-old woman with history of an abnormal pap smears who presented with a large pelvic mass with initial biopsy of cervix demonstrating squamous cell carcinoma with negative PDL1 expression and a CPS score of 0. She underwent chemoradiation and presented three months after primary treatment completion with recurrence of squamous cell carcinoma and positive PD-L1 expression with a CPS score of 20. Pembrolizumab was added to cycle three of her systemic chemotherapy regimen of carboplatin/paclitaxel/bevacizumab. She had progression on this regimen and was transitioned to tisotumab vedotin; however, ultimately opted to proceed with hospice secondary to failure to thrive.

Case 2

A 36-year-old woman with history of an abnormal pap smear in pregnancy and initial biopsy demonstrating endocervical adenocarcinoma, mucinous type. She underwent open radical hysterectomy, bilateral salpingectomy, bilateral oophorepexy, and bilateral pelvic lymph node dissection with subsequent adjuvant chemoradiation. Her initial pathology demonstrated positive PDL1 expression with CPS score of 15. She presented six months after completion of primary treatment with recurrence of endocervical adenocarcinoma, mucinous type and negative PD-L1 expression with a CPS score of < 1. Regardless of this discrepancy, pembrolizumab was added to cycle five of her systemic chemotherapy regimen of carboplatin/paclitaxel/bevacizumab. She initially demonstrated a mixed response; however, ultimately progressed after eight cycles and was transitioned to tisotumab vedotin.

**Discussion:**

To our knowledge, discrepancies in PD-L1 expression in a matched setting between primary and metastatic tumors has only been reported once. This is the first case report describing these inconsistencies. Etiologies of and outcomes related to the discrepant expression of PD-L1 should be further studied.

## Highlights

• PD-L1 expression and subsequent combined positive score (CPS) is used to determine eligibility for Pembrolizumab in recurrent cervical cancer.
• In clinical practice, the CPS score is usually tested once on the initial tumor and not again in the recurrent specimen.
• Discrepancies in PD-L1 expression between primary and metastatic tumor site have been observed in various other cancers.
• Two cases of recurrent cervical cancer with discordance in PD-L1 expression have been identified.

## 1 Introduction

Despite advances in screening and vaccination programs, cervical cancer continues to be a global epidemic. For patients with early-stage cervical cancer, prognosis is excellent after appropriate surgical intervention with the five-year overall survival approaching 85 %; however, for patients with advanced stage or recurrent cancer, the prognosis is poor and effective treatment options are limited (Chen et al., 2015).

Substantial progress has been made utilizing immunotherapy in cervical cancer—specifically using checkpoint inhibition to impede immune inhibitory pathways such as programmed cell death 1 (PD-1)/PD-1 ligand (PD-L1). It has been demonstrated that persistent HPV infection upregulates PD-L1 expression in cervical cancer (Liu et al., 2016). PD-L1 positivity has been identified in both squamous cell carcinoma (SCC) and adenocarcinoma of the cervix; however, it is more commonly identified in SCC. In patients with SCC of the cervix, PD-L1 has been linked to poorer disease-free survival (PFS) and overall survival (OS) in patients with diffuse expression compared to marginal expression (Heeren et al., 2016). Pembrolizumab is a monoclonal antibody directed against PD-L1 and has been approved by the FDA for recurrent cervical cancer patients who have a combined positive score (CPS) > 1. In clinical practice, the CPS score is usually tested once on the initial tumor and not again in the recurrent specimen. We report two cases in which the expression of PD-L1 differed between primary and recurrent cervical cancer tumor and the use of pembrolizumab in these two patients.

### 1.1 Case 1

A 42-year-old woman presented to the emergency department in October of 2022 after a large 10 cm x 9 cm x 8 cm pelvic mass was seen on an ultrasound by her primary care physician. She had a personal history of two abnormal pap smears during pregnancy but was lost to follow up due to insurance concerns. Patient reported slow intermittent vaginal bleeding over the course of four months, vaginal pressure, and a forty-pound weight loss. Her presenting hemoglobin was 7.9. A biopsy of the cervical mass was performed by a gynecologic oncologist which demonstrated moderate to poorly differentiated squamous cell carcinoma with necrosis. Immunohistochemistry demonstrated the tumor infiltrating as a nest of polygonal cells with abundant eosinophilic cytoplasm and pleomorphic nuclei with scattered dyskeratotic cells. HPV association and p16 staining were not completed for this specimen. The PD-L1 score from her initial biopsy was negative with a combined positive score of 0.

In addition to a pelvic exam, multiple modalities of imaging were performed to determine stage including computerized tomography (CT), positron emission tomography (PET)/CT, and magnetic resonance imaging (MRI) all of which confirmed International Federation of Gynecology and Obstetrics (FIGO) stage IIIC1 cervical cancer by demonstrating a large soft tissue mass centered in the cervix extending into the lower third of the vagina as well as enlarged likely metastatic pelvic lymph nodes.

She was treated with primary chemoradiation – weekly cisplatin 40 mg/m2 with radiation for six weeks. She received a total of 4500 cGy of external beam radiation therapy delivered in 25 equal fractions to the pelvis. Her follow up MRI demonstrated improvement of the cervical lesion and no adenopathy. This was then followed with two, high dose rate (HDR) brachytherapy procedures in which three HDR fractions of brachytherapy were administered for a total dose of 1500 per procedure at 500 cGy per fraction. Plan at this time was to complete PET/CT scan in three months without plans for additional chemotherapy or immunotherapy prior to repeat scans.

**Fig. 1:**
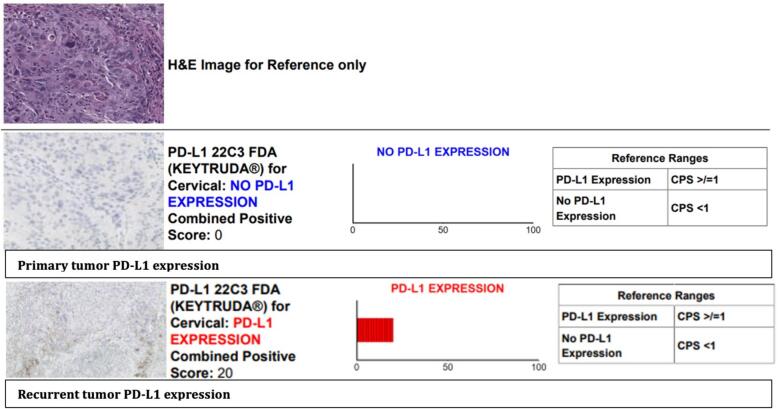
Immunohistochemistry showing comparison of PD-L1 expression in primary and metastatic tumor sites for case 1.

Three months after primary treatment completion, she presented to a family medicine clinic for three weeks of cough, left-sided swollen lymph node of the neck, fatigue, and body aches. She had a PET scan demonstrating persistent FDG uptake in a smaller mass within the cervix and slightly smaller pelvic lymph nodes, but with evidence of new distant metastatic disease in the left supraclavicular nodes, mediastinal nodes, retrocural nodes, paraortic nodes, porta hepatis nodes, and metastatic left lung nodules. An interventional radiology (IR) guided biopsy of the left neck mass was performed and confirmed recurrence of squamous cell carcinoma. Immunohistochemistry demonstrated moderately to poorly differentiated squamous cell carcinoma involving fibroconnective tissue. HPV association and p16 staining were not completed for this specimen. She was next treated with systemic chemotherapy; carboplatin/ paclitaxel/bevacizumab. A PD-L1 score was requested from the metastatic supraclavicular node and was found to have a CPS of 20 for PD-L1 (Fig. 1). Given the change in PD-L1 expression, she was considered a candidate for pembrolizumab which was added to cycle three of her chemotherapy regimen.

She subsequently developed a left internal jugular vein thrombus and left arm weakness requiring admission to the hospital and palliative radiation to her supraclavicular lymph node. She was readmitted to the hospital for altered mental status a few weeks later with a negative workup, possibly due to opioid withdrawal. During this hospitalization, her CT scan demonstrated progression of disease with new hydronephrosis and pelvic fluid and so her chemotherapy regimen was changed to tisotumab vedotin. Additionally, she was found to have a new inferior vena cava clot while on Lovenox. She received one cycle of tisotumab vedotin, but two weeks later opted to proceed with hospice secondary to failure to thrive.

### 1.2 Case 2

In August of 2022, a 36-year-old woman presented four weeks after cesarean section for initial consultation after cervical biopsy in the third trimester demonstrated endocervical adenocarcinoma. She had initially noticed spotting during pregnancy, and a pap smear resulted in ASCUS/hrHPV+. She underwent cervical biopsies in her late third trimester that returned endocervical adenocarcinoma, mucinous type. Her postpartum course was complicated by multiple emergency department visits for heavy vaginal bleeding requiring blood transfusion. At her initial consultation visit, a two centimeter, firm lesion was noted on her posterior cervix without parametrial involvement. She was diagnosed with FIGO stage 1B1 endocervical adenocarcinoma. She had a normal pap smear one year prior to her abnormal pap smear. She underwent PET/CT scan that showed no evidence of distant disease. She subsequently underwent open radical hysterectomy, bilateral salpingectomy, bilateral oophoropexy, and bilateral pelvic lymph node dissection. Post-operative tumor board review confirmed Stage 1B3 adenocarcinoma with lymphovascular space invasion. Her final tumor size was 4.9 cm with invasion into the deep 1/3 of stroma, negative margins, and had two of 23 lymph nodes positive for carcinoma. The decision was made to proceed with adjuvant chemoradiation.

She was treated with primary chemoradiation – weekly cisplatin 40 mg/m2 with radiation for six weeks. She received a total of 5040 cGy of external beam radiation therapy delivered in 28 equal fractions to the pelvis. Her follow up PET/CT 3 months after completion demonstrated low level activity at midline anterior pelvic scar, likely post-surgical. Plan at this time was to complete PET/CT scan in three months without plans for additional chemotherapy or immunotherapy prior to repeat scans.

Six months after completion of primary treatment, the patient was complaining of midline abdominal pain near her previous incision. PET/CT demonstrated multiple new FDG avid low pelvic subcutaneous and intramuscular soft tissue masses. CT demonstrated a 2.3 cm necrotic mass at the lower anterior abdominal wall with soft tissue stranding and skin thickening, and sub adjacent additional nodules measuring up to 2.3 cm. IR biopsy was completed of the abdominal wall nodule demonstrating recurrence of endocervical adenocarcinoma, mucinous type, HPV-associated. Immunohistochemistry testing demonstrated the tissue to be MUC4 and p16 positive, and ER/PR negative. PDL-1 testing was sent one week after the biopsy was collected. The decision was made to proceed with systemic chemotherapy, carboplatin/paclitaxel/bevacizumab.

**Fig. 2:**
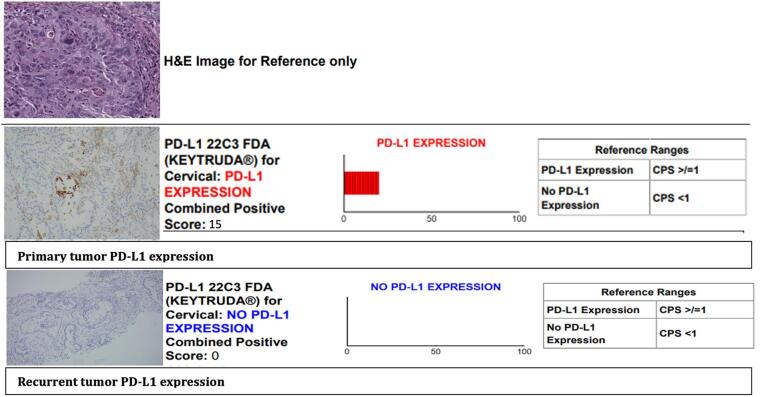
Immunohistochemistry showing comparison of PD-L1 expression in primary and metastatic tumor sites for case 2.

Two months after starting chemotherapy, she had a repeat PET/CT scan demonstrating partial metabolic response to therapy with decreased FDG uptake and size of metastases in the midline abdominal wall incision site and near complete resolution of previously seen FDG avid foci adjacent the rectus abdominus muscles with only one residual metastasis with mild FDG uptake. Her CPS score from her primary surgery was 15, but her CPS score from biopsy at abdominal wall recurrence site was < 1. (Fig. 2) Regardless of the discrepancy, pembrolizumab was added to cycle five of the patient’s treatment regimen.

After three months and four cycles of pembrolizumab to the existing treatment regimen, a repeat PET/CT demonstrated a mixed response with increased metabolic activity and size of midline abdominal wall metastatic lesions, and decreased size and metabolic activity of some lower ventral abdominal wall and anterior mesenteric lesions. After two months and 11 cycles of carboplatin/paclitaxel/bevacizumab and seven cycles of pembrolizumab, repeat PET/CT demonstrated interval increase in size and uptake of the soft tissue nodules in the anterior abdominal wall along the anterior pelvic peritoneum with focal uptake in the left external iliac region. She underwent 12 cycles of carboplatin/paclitaxel/bevacizumab and eight cycles of pembrolizumab while awaiting imaging results. She underwent repeat biopsy to test for HER2, which was found to be equivocal. She was consented for tisotumab vedotin and is scheduled to begin cycle one pending her baseline ophthalmologic exam.

## 2 Discussion and conclusions

The role of PD-L1 in a tumor’s ability to evade normal immune surveillance has made it a key target for immunotherapy. Pembrolizumab was approved in 2018 for use in advanced stage cervical cancer with positive PD-L1 expression based on results from Keynote 158(Schellens et al., 2017). Keynote 158 demonstrated an increased objective response rate and tolerable adverse effect profile in patients given pembrolizumab who had PD-L1 positive tumors (CPS>1). Additional studies have demonstrated improved progression free and overall survival in patients with PD-L1 positive tumors receiving pembrolizumab compared to placebo in combination with chemotherapy +/- bevacizumab (Colombo et al., 2021). Recently, the FDA approved pembrolizumab as part of the initial treatment course for metastatic cervical cancer with study A18; agnostic to CPS score (Lorusso et al., 2020).

It is current clinical practice that primary tumors undergo testing for PD-L1 expression to assess for eligibility for pembrolizumab in the recurrent setting. The National Comprehensive Cancer Network (NCCN) does not describe re-testing recurrent tumor samples for PD-L1 in cervical cancer when the initial tumor has a negative score. In our two cases, PD-L1 expression differed between primary and metastatic tumor site when retested. Pembrolizumab was offered to both patients regardless of the discrepancy. Although one patient did not have a favorable response, the other initially demonstrated a mixed response which was then followed by progression. The difference in response to treatment in those who receive pembrolizumab might be due to the differences in expression level of PD-L1 among tumors in different locations, presence of absence of tumor necrosis, and/or impact of treatment regimen (e.g radiation, chemotherapy).

Incongruity in PD-L1 expression between primary and metastatic tumor site has been observed in a variety of cancers including clear-cell renal cell carcinoma, non-small cell lung carcinoma, melanoma, breast, and ovarian cancer which has led to re-testing of PD-L1 expression in patients with recurrent and/or metastatic disease (Callea et al., 2015, Munari et al., 2018, Yuan et al., 2019, Madore et al., 2015, Parvathareddy et al., 2021). These discrepancies have not been well studied in the setting of cervical cancer and to our knowledge only one prior study has investigated these differences in a matched setting. Liu et al. demonstrated a 33 % discrepant rate of PDL-1 expression in primary versus recurrent/metastatic matched samples with a lower rate of PD-L1 positivity identified in primary lesions compared to recurrent/metastatic lesions (15.4 % vs 30.4 %) (Liu et al., 2023).

The etiology of these discrepancies has yet to be identified. A possible explanation is a change of the tumor microenvironment in response to neoadjuvant chemotherapy (Liang et al., 2020, Meng et al., 2018). Additionally, it has been previously reported that PD-L1 expression can differ secondary to biopsy site (Liu et al., 2023, Huang et al., 2021); however, this is up for debate as a separate study demonstrated no significant difference when comparing biopsies of primary tumor to metastatic lymph nodes (Heeren et al., 2016). Given no prospective study has been performed, the preservation time prior to performing PD-L1 analysis is also a factor that may confound these results.

Disease outcomes in patients with discrepant expression of PD-L1 in cervical cancer should be further studied. Additionally, genetic drift amongst synchronous lesions under the influence of intervening therapy and of different metastatic sites requires further study.

## Author contributions

BF: Conceptualized topic, investigation of topic, and wrote the original draft of manuscript. AH: Conceptualized topic, contributed to discussion, and provided senior guidance and supervision. All authors approved the final version of the manuscript.

## CRediT authorship contribution statement

**Brittany File:** Writing – review & editing, Writing – original draft, Conceptualization. **Anjali Hari:** Writing – review & editing, Writing – original draft, Supervision, Conceptualization.

## Declaration of competing interest

The authors declare that they have no known competing financial interests or personal relationships that could have appeared to influence the work reported in this paper.
